# Gellan gum spongy‐like hydrogel‐based dual antibiotic therapy for infected diabetic wounds

**DOI:** 10.1002/btm2.10504

**Published:** 2023-03-21

**Authors:** Ana Isabel Mendes, Alexandra Gabriel Fraga, Maria João Peixoto, Ivo Aroso, Adhemar Longatto‐Filho, Alexandra Pinto Marques, Jorge Pedrosa

**Affiliations:** ^1^ Life and Health Sciences Research Institute (ICVS) School of Medicine University of Minho Braga Portugal; ^2^ ICVS/3B's–PT Government Associate Laboratory Braga/Guimarães Portugal; ^3^ 3B's Research Group, I3Bs – Research Institute on Biomaterials, Biodegradables and Biomimetics Headquarters of the European Institute of Excellence on Tissue Engineering and Regenerative Medicine University of Minho Guimarães Portugal; ^4^ Molecular Oncology Research Center Barretos Cancer Hospital Barretos São Paulo Brazil; ^5^ Laboratory of Medical Investigation (LIM) 14 Hospital das Clínicas da Faculdade de Medicina da Universidade de São Paulo São Paulo Brazil

**Keywords:** antibiotics, diabetic ulcers, hydrogel, MRSA infection, topical delivery

## Abstract

Diabetic foot infection (DFI) is an important cause of morbidity and mortality. Antibiotics are fundamental for treating DFI, although bacterial biofilm formation and associated pathophysiology can reduce their effectiveness. Additionally, antibiotics are often associated with adverse reactions. Hence, improved antibiotic therapies are required for safer and effective DFI management. On this regard, drug delivery systems (DDSs) constitute a promising strategy. We propose a gellan gum (GG)‐based spongy‐like hydrogel as a topical and controlled DDS of vancomycin and clindamycin, for an improved dual antibiotic therapy against methicillin‐resistant *Staphylococcus aureus* (MRSA) in DFI. The developed DDS presents suitable features for topical application, while promoting the controlled release of both antibiotics, resulting in a significant reduction of in vitro antibiotic‐associated cytotoxicity without compromising antibacterial activity. The therapeutic potential of this DDS was further corroborated in vivo, in a diabetic mouse model of MRSA‐infected wounds. A single DDS administration allowed a significant bacterial burden reduction in a short period of time, without exacerbating host inflammatory response. Taken together, these results suggest that the proposed DDS represents a promising strategy for the topical treatment of DFI, potentially overcoming limitations associated with systemic antibiotic administration and minimizing the frequency of administration.

## 1 INTRODUCTION

Diabetes is a serious condition that affects 537 million people worldwide, expected to rise to 783 million people by 2045.^1^ This chronic metabolic disease constitutes a powerful risk factor to foot ulceration, which is highly linked to physiological alterations underlying diabetes, such as peripheral vascular disease, neuropathy and impaired immunity, that collectively contribute to a microenvironment favorable to bacterial growth and infection.^2^, ^3^, ^4^, ^5^

Diabetic foot infection (DFI) represents a significant source of morbidity and is associated with an increased risk of limb amputation.^6^ Most of DFIs are confined to skin, but due to vascular insufficiency and abnormal immune response, microorganisms can spread to deeper tissues, including tendons, fascia, muscle, joints and bone.^7^, ^8^ Multiple bacterial species are often found in DFI, from which *Staphylococcus aureus* predominates as the most frequently isolated and virulent pathogen.^5^, ^7^, ^9^, ^10^ Methicillin‐resistant *Staphylococcus aureus* (MRSA), in particular, represents a significant health threat in hospital and community settings, with a prevalence ranging 15%–40%.^11^, ^12^ A wide range of antibiotics of different classes has been used for DFI treatment, isolated or combined.^12^, ^13^ For MRSA infections, vancomycin (VAN) and clindamycin (CLD) represent two frequently used antibiotics.^13^, ^14^, ^15^ VAN is a glycopeptide administrated parenterally (500–1000 mg IV every 12 h) due to its poor bioavailability. Although some isolates of VAN resistant *S. aureus* have recently emerged, this antibiotic remains one of the first choices for severe MRSA infections.^16^ CLD belongs to the class of lincosamides and is one of the first line antibiotics for the treatment of mild and moderate DFI, either oral (PO) or intravenous (IV) route (600–900 mg PO every 6 h, or 600–2400 mg IV once daily).^12^, ^13^ CLD can also be used to treat severe DFI, although in these cases it should be combined with other antibiotics, such as VAN.^13^

Although crucial to treat DFI, antibiotic therapy faces some challenges and drawbacks. In the context of DFI, one of the most important factors limiting antibiotic therapy is the peripheral vascular disease that hinders antibiotic delivery and penetration into infected tissues at effective concentrations. Consequently, pharmacological activity of antibiotics can be compromised, resulting in failure to treat DFI, as well as an increased risk of emergence of antibiotic resistance.^7^ Another limitation of antibiotic therapy is the occurrence of adverse effects.^17^, ^18^, ^19^, ^20^

Therefore, new and more effective therapeutic strategies are needed to improve antibiotic therapy in eradicating complicated DFI caused by MRSA, ensuring therapeutic concentrations at the target site, while minimizing potential systemic adverse effects and the risk of bacterial resistance. In this regard, drug delivery systems (DDSs) have evolved as an alternative strategy for circumventing the constraints of conventional therapy. Furthermore, different structures of DDS have been proposed in the literature, for example, particulate systems,^21^, ^22^ nanofibers,^23^ hydrogels,^24^, ^25^, ^26^, ^27^, ^28^, ^29^ polymer–drug conjugates,^30^ according to the physicochemical properties of the drug and to grant the benefits of the intended therapeutic administration route. Here, we propose an alternative therapeutic approach for MRSA‐infected diabetic wounds, based on a topical administration of VAN and CLD using gellan gum (GG)‐based spongy‐like hydrogels. GG is an extracellular bacterial polysaccharide that naturally forms thermoreversible physical hydrogels and is achieving promising results for drug delivery^31^, ^32^, ^33^, ^34^, ^35^, ^36^, ^37^ and wound healing purposes.^38^, ^39^, ^40^, ^41^ In particular, GG‐based spongy‐like hydrogels possess unique features that can benefit the wound healing process, including (i) improved mechanical properties which grant wound adaptability and manipulation without break; (ii) water retention capability allowing wound exudate absorption and moisture retention for wound hydration; (iii) act as a regenerative template.^38^, ^42^, ^43^ These features combined with an off‐the‐shelf availability and a simple method of production, turns GG‐based spongy‐like hydrogels an appealing DDS in the context of local antibiotic therapy.

Presently, we developed GG‐based formulations loading VAN and CLD that possess suitable physicochemical properties for a controlled and topical delivery of both antibiotics. The antibacterial activity of loaded GG‐based spongy‐like hydrogels against MRSA, as well as their potential to reduce antibiotic toxicity were firstly assessed in vitro and further corroborated in vivo using a diabetic mouse model of MRSA‐infected wounds. Proposed formulation showed a good antibacterial activity, while significantly reduced the intrinsic antibiotic cytotoxicity without aggravating the host inflammatory response.

## 2 RESULTS

### 2.1 In vitro antibacterial effect of interaction between VAN and CLD

**FIGURE 1:**
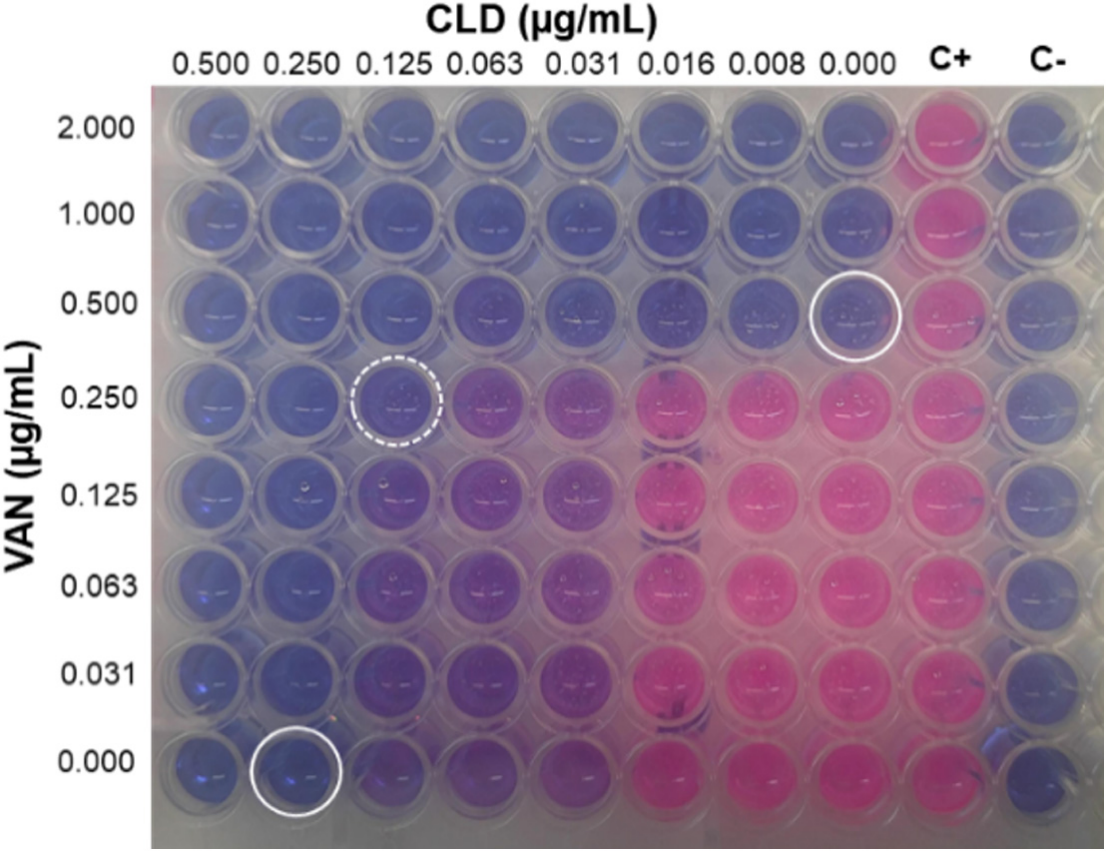
Representative result of the checkerboard assay of VAN and CLD against MRSA. Resazurin reduction to resofurin by metabolically active bacteria is indicated by color transition from blue to pink. Close circles indicate the MIC of VAN and CLD when tested alone and open circle shows the MIC of their combination. C+ corresponds to positive control wells (bacteria) and C− corresponds to negative control wells (medium).

We first conducted a checkerboard assay to confirm the susceptibility of MRSA strain to VAN and CLD and evaluate the effect of VAN/CLD interaction on antibacterial activity. A representative result is shown in Figure 1, demonstrating the MIC of VAN at 0.5 μg/mL and the MIC of CLD at 0.25 μg/mL. According to Clinical Laboratory Standard Institute (CLSI),^44^ these MIC are indicative of a susceptible bacterial strain to both antibiotics. Furthermore, when tested in combination, the MIC of VAN and CLD was halved, suggesting an additive interaction effect determined by a fractional inhibitory concentration (FICI) = 1.

### 2.2 Physicochemical properties of loaded GG‐based formulations

**FIGURE 2:**
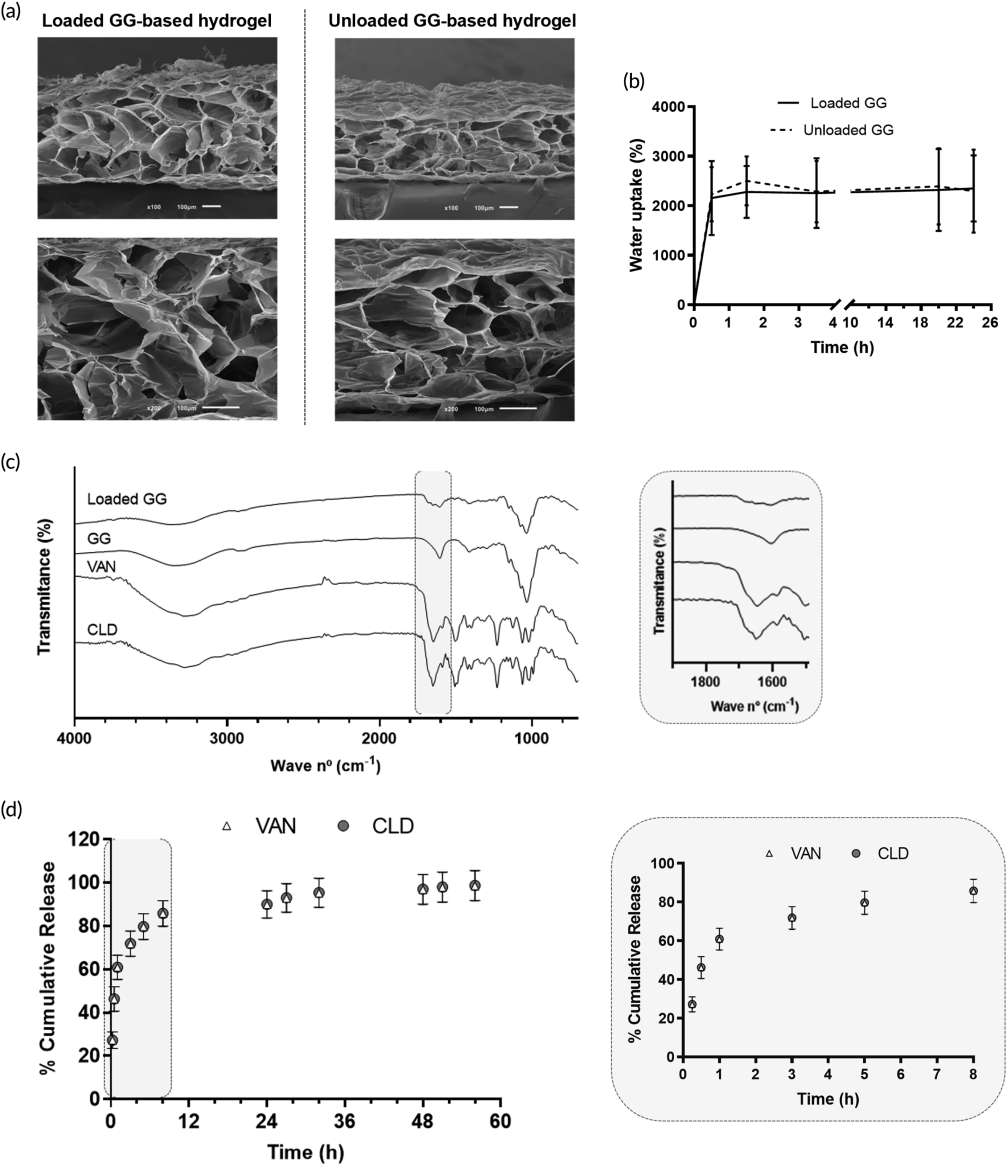
Physicochemical properties of GG‐based formulations: (a) representative scanning electron microscope (SEM) micrographs of the internal morphology of loaded and unloaded GG‐based structures (scale bar 100 μm); (b) water uptake capacity, in %, overtime; (c) IR spectra of loaded GG‐based structures and raw materials. Region of the characteristics peaks of VAN and CLD is highlighted and zoomed in gray box; (d) profile of in vitro VAN and CLD cumulative release (%). Gray box represents a zoomed overview of the release profile within the first 8 h.

Given the additive effect of VAN and CLD against MRSA, we next characterized the structure of loaded GG‐based structures to assess the impact of antibiotic incorporation on the physical properties of these formulations and to identify any potential polymer‐antibiotics interactions. Loaded GG‐based structures revealed a porous and crosslinked internal network, similar to unloaded structures (Figure 2a) that, upon rehydration, granted a water uptake capacity superior to 2000%, within the first 30 minutes of incubation. This uptake capacity remained stable through the following incubation time (Figure 2b), without significant differences when compared to unloaded GG‐based spongy‐like hydrogels (*p* > 0.05).

Fourier transform infrared spectroscopy‐attenuated total reflectance (FTIR‐ATR) analysis of loaded GG‐based structures (Figure 2c) revealed spectral peaks characteristic of antibiotics and polymer, with no alterations in the characteristic peaks or in the appearance of new functional groups. Specifically, the infrared (IR) spectrum of loaded GG‐based structures showed small peaks in the range of 1700–1600 cm^−1^ related to the C=O stretching of amide carbonyl group of VAN^45^ and CLD,^46^, ^47^ in addition to the characteristic peaks of GG: at around 3370 cm^−1^ attributed to O—H stretching of hydroxyl group; at 2914 cm^−1^ due to C—H stretching of alkyl group; at 1605 cm^−1^ related to the C=O stretching of carboxylate group; at 1405 cm^−1^ of methyl C—H bending; and at 1040 cm^−1^ attributed to the C—O stretching of alkyl ether.^48^, ^49^, ^50^

The release profile of VAN and CLD from GG‐based spongy‐like hydrogels was characterized by a burst release within the first 8 h of incubation, followed by a sustained release (Figure 2d). Considering the maximum experimental mass per loaded GG‐based spongy‐like hydrogel, respectively 32.6 and 42.3 μg for VAN and CLD, the cumulative release profile was identical for both antibiotics. Approximately 60.8% of VAN and 61.0% of CLD was released within the first hour, reaching respectively 85.7% and 85.9% of the incorporated amount after 8 h. The remaining antibiotics were further released in a sustained manner until the endpoint. The best model fitting the cumulative release data was the Korsmeyer‐Peppas, with a *R* ^2^_adjusted_ of 0.836 and 0.867 for VAN and CLD, respectively, and a release exponent (*n*) inferior to 0.45 for both antibiotics.

### 2.3 In vitro antibacterial activity and cytocompatibility of loaded GG‐based spongy‐like hydrogels

**FIGURE 3:**
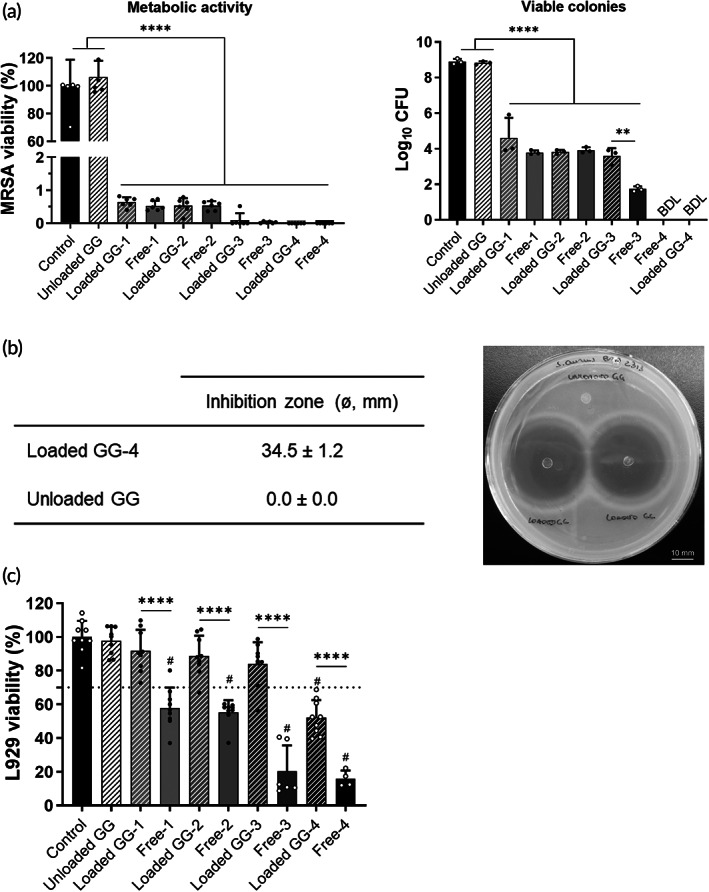
In vitro biological activity of loaded GG‐based spongy‐like hydrogels: (a) antibacterial effect using the broth dilution assay, expressed as bacterial metabolic activity (left) and growth of viable colonies (right). BDL: below detection limit; (b) bacterial inhibition zone from the agar diffusion assay, expressed in mm (left) and represented by a tested plate showing two replicates of loaded GG‐4 (right). Scale bar corresponds to 10 mm; (c) cytotoxic effect in L929 cells. **p < 0.01; ****p < 0.0001; ^#^ *p* < 0.0001 in comparison to control.

We then evaluated the suitability of the attained release profile over the in vitro antibacterial activity of loaded GG‐based spongy‐like hydrogels against MRSA. Different concentrations of antibiotics were tested using the broth dilution assay, to assess a possible dose–response effect (Table 1). Figure 3a shows that all tested concentrations of loaded antibiotics resulted in a complete inhibition of metabolic viability of MRSA, in addition to a drastic reduction of bacterial replication capacity. In this regard, loaded GG‐based spongy‐like hydrogels‐1 to ‐3 led to approximately half decay of Log_10_ colony forming units (CFU) in comparison to control, while no bacterial growth was observed for loaded GG‐based spongy‐like hydrogel‐4. Moreover, in comparison to the corresponding free VAN/CLD solutions, loaded GG‐based spongy‐like hydrogels presented a similar antibacterial activity, except for loaded GG‐based spongy‐like hydrogel‐3, in which a higher bacterial growth inhibition was observed for free VAN/CLD solution (*p* < 0.01). Moreover, unloaded GG‐based spongy‐like hydrogels did not present any antibacterial effect (*p* > 0.05 compared to control). The antibacterial effect of loaded GG spongy‐like hydrogels‐4 was further confirmed in an agar diffusion assay. A mean inhibition zone of 34.5 mm was observed (Figure 3b), in opposition to its absence for unloaded GG‐based spongy‐like hydrogels (0.0 mm), in agreement with the lack of antibacterial effect found in the broth antibacterial assay.

**TABLE 1:** Final concentrations of VAN and CLD incorporated in loaded GG‐based hydrogels.

	VAN (μg/mL)	CLD (μg/mL)
Loaded GG‐based hydrogel‐1	2.5	3.3
Loaded GG‐based hydrogel‐2	5.0	6.5
Loaded GG‐based hydrogel‐3	50.0	65.0
Loaded GG‐based hydrogel‐4	2000.0	2600.0

Furthermore, in vitro cytocompatibility of loaded GG‐based hydrogels was assessed in L929 fibroblasts, to determine the potential of developed DDS in reducing free antibiotic‐associated toxicity. Viability of cells treated with loaded GG‐based spongy‐like hydrogels‐1 to ‐3 was superior to 80%, while loaded GG‐based spongy‐like hydrogel‐4 reduced cell viability in about 50% (Figure 3c). All tested free VAN/CLD solutions reduced cell viability by more than 40%, and this effect increased as higher concentrations were tested. Treatment with unloaded GG‐based spongy‐like hydrogels resulted in a cell viability similar to untreated cells (control).

### 2.4 Biological effect of loaded GG‐based spongy‐like hydrogels in a diabetic mouse model of MRSA‐infected wounds

Considering the antibacterial activity and cytocompatibility of loaded GG‐based spongy‐like hydrogels, we next assessed the effect of these formulations in controlling infection and supporting wound healing in vivo, using an optimized diabetic mouse model of MRSA‐infected full‐thickness excisional wounds.

**FIGURE 4:**
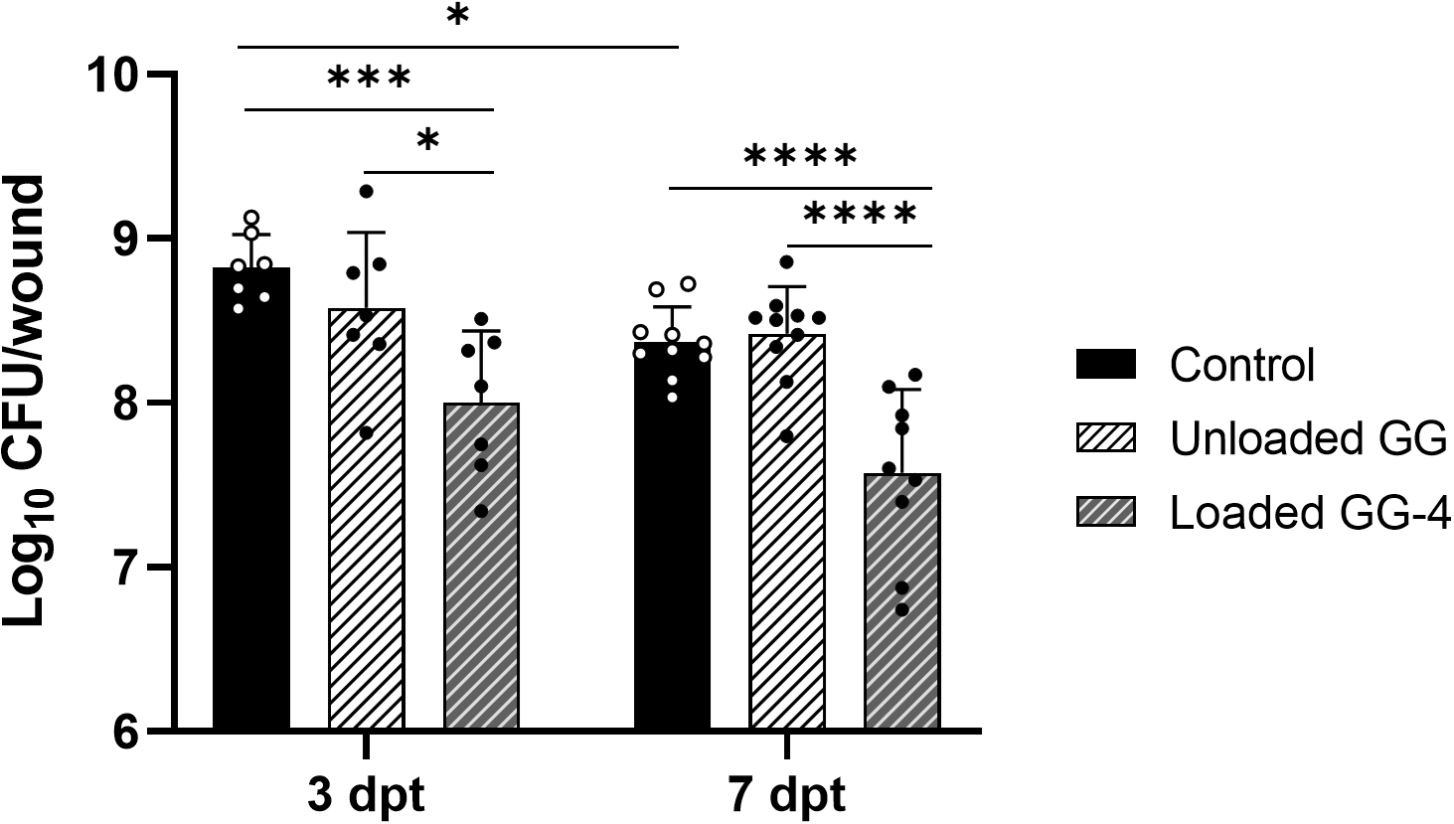
Quantitative bacteriological data of MRSA‐infected wounds of diabetic mice assessed after 3 and 7 days of a single administration of loaded GG‐based spongy‐like hydrogel‐4, in comparison to unloaded GG and control groups. *p < 0.05; ***p < 0.001; ****p < 0.0001.

Treatment was performed with GG‐based spongy‐like hydrogel‐4 that showed significant in vitro antibacterial activity, while not compromising cytocompatibility. Treating infected wounds with a single administration of loaded GG‐based spongy‐like hydrogel led to a significant reduction of bacterial burden (approximately one Log_10_) in comparison to the other experimental groups (unloaded GG‐based spongy‐like hydrogels and control) (Figure 4). Also, from day 3 to day 7 post‐treatment, there was a slight reduction on the bacterial counts of wounds for all experimental groups, though statistical significance was only found for the control group (*p* < 0.05).

**FIGURE 5:**
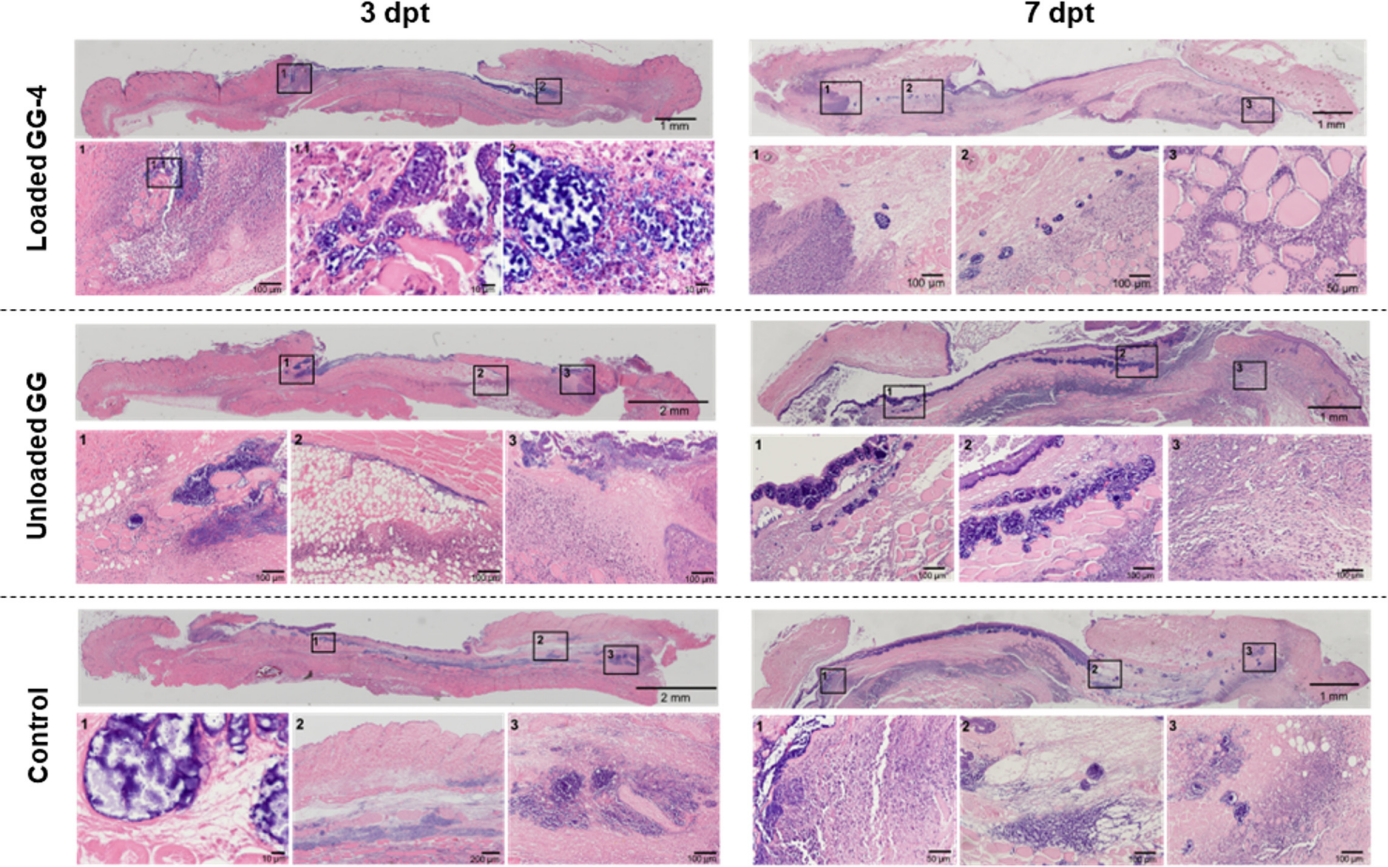
Representative micrographs of wound sections stained with H&E, of the three experimental groups at 3 dpt (left) and 7 dpt (right) of a single administration of loaded GG‐based spongy‐like hydrogel‐4, in comparison to unloaded GG‐based spongy‐like hydrogel and control groups. Boxes represent areas of high magnification at the lower panel.

The results from quantitative bacteriological evaluation were also complemented by histopathological analysis. At 3 dpt, all groups showed an established wound infection (Figure 5), with bacteria covering not only the wound surface, but also penetrating deeply into the wound bed. This was noticeably less evident for treatment groups than for the control and unloaded GG‐based spongy‐like groups, in which several clusters of bacteria were found beneath the wound surface and also spread into the non‐wounded dermis and subcutaneous tissue at the wound margin. Moreover, the wounded area in all experimental groups showed tissue liquefaction necrosis, edema, and inflammation, characterized by the infiltration of inflammatory cells, predominantly neutrophils. At day 7 post‐treatment, infection was still highly present, as indicated by the visible clusters of bacteria and large amounts of inflammatory cells surrounding the clusters. Extensive areas of necrosis and edema were still identified at 7 dpt (Figure 5). Furthermore, wounds remained open throughout the experimental period, regardless the experimental group, without significant signs of granulation tissue formation and reepithelization.

**FIGURE 6:**
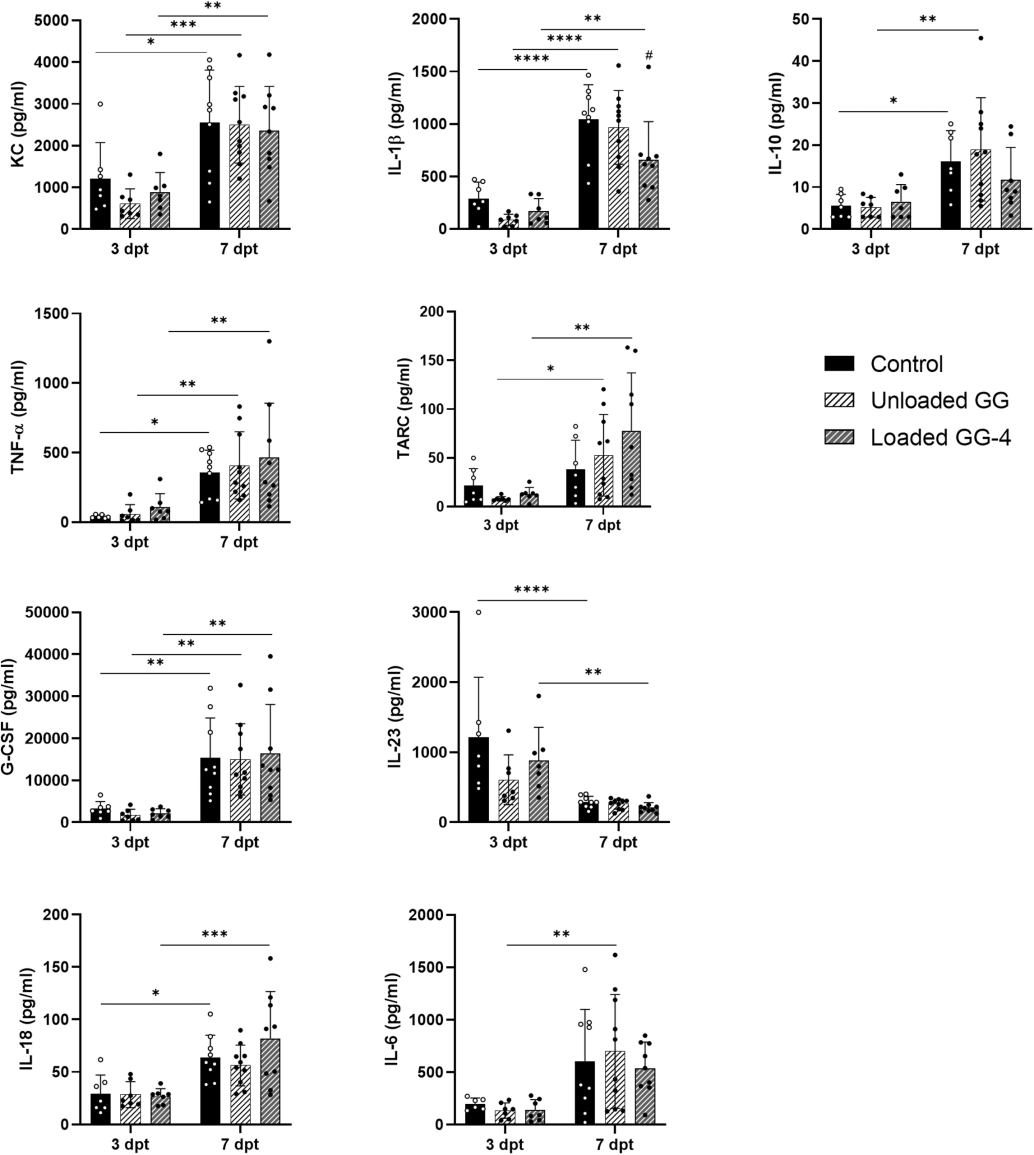
Tissue levels of pro‐inflammatory (KC, IL‐18, IL‐23, TNF‐α, IL‐1β, IL‐6) and anti‐inflammatory (G‐CSF, TARC, IL‐10) mediators from diabetic mouse model of MRSA‐infected wounds after 3 and 7 days of treatment. *p < 0.05; **p < 0.01; ***p < 0.001; ****p < 0.0001; ^#^ *p* < 0.0001 in comparison to unloaded GG and control groups at 7 dpt.

Immunological analysis was also performed to evaluate a possible effect of the treatment on the immunomodulation of the host defense. Several cytokines and chemokines were quantified in the wound tissue as well as in the serum. Overall, tissue analysis revealed an increment of most of the tested immune mediators from 3 to 7 dpt and no significant differences between treatments were observed, except for the pro‐inflammatory cytokine IL‐23 (Figure 6). In this case, a significant decrease was observed for the group treated with loaded GG‐based spongy‐like hydrogel in relation to the unloaded GG‐based spongy‐like hydrogel and control groups (*p* < 0.05). Regarding the levels of immune mediators in the serum (Figure S1), these remained relatively stable between timepoints and a similar profile was observed between the different experimental groups, noticing only a significant difference for G‐CSF at 3 dpt between loaded GG‐based spongy‐like hydrogel and control (*p* < 0.01). Levels of lL‐10 were undetectable in the serum at both timepoints.

## 3 DISCUSSION

One of the most common complications arising from diabetes is DFI, in which *S. aureus* is frequently the causative pathogen. Treating *S. aureus* infections has been increasingly challenging, owing the ability of this pathogen to develop resistance to several antibiotics of choice.^51^ Indeed, the emergence of antibiotic resistance is an important public health issue that has been threatening the efficacy of currently available antibiotics. Moreover, in the past years, new classes of antibiotics have been hard to find in the market, given the drastic reduction of investment of the pharmaceutical industry on antibiotic research due to scientific, regulatory, and financial constraints.^52^, ^53^ Under these circumstances, combination therapy using two or more antibiotics has been an attractive approach for difficult‐to‐treat bacterial infections, minimizing the risk of antibacterial resistance and potentially contributing to an increased drug efficacy.^54^ Therefore, our therapeutic approach considered a combination therapy based on two antibiotics, namely VAN and CLD, often used in the clinical management of MRSA in DFI. It is reported that VAN inhibits bacterial cell wall synthesis,^19^, ^20^, ^55^, ^56^ while CLD disrupts bacterial protein synthesis,^17^, ^18^, ^56^ and these mechanisms of action showed herein to have an additive interaction against MRSA, supporting the benefit of the proposed combination therapy.

Further incorporation of these antibiotics in a DDS suitable for local application, as GG‐based spongy‐like hydrogels, can contribute to circumvent subtherapeutic concentrations of systemic administration, that arise from DFU pathophysiology and have been also associated with the emergence of resistant bacterial strains.^13^, ^57^ The in vitro characterization of the developed loaded GG‐based spongy‐like hydrogel confirmed that the incorporation of VAN and CLD did not disturb the intrinsic physicochemical properties of this DDS, that was able to release both antibiotics in a controlled pattern governed by diffusion. The reported release kinetics is a valuable approach for treating infection locally, since an initial high dose can lead to bacterial elimination, while the further sustained release will prevent bacterial regrowth and biofilm rebuilding.^58^ This is consistent with previous studies on GG‐based antibiotic delivery systems reporting the release of entrapped molecules through a biphasic profile (initial burst phase followed by a long‐sustained phase), while ensuring good antibacterial performances of released antibiotics.^59^, ^60^ While DDS degradation can determine their therapeutic performance, it is not a factor to consider in our system since GG‐based spongy‐like hydrogels remain stable in saline at 37°C for at least 28 days, with residual mass loss.^43^, ^61^, ^62^ Furthermore, as the potential clinical application of the proposed formulation is not expected to exceed 3 days (considering the antibiotic release profile and frequency of wound care procedures), hydrogel degradation would not likely affect treatment efficacy.

Notably, the reported controlled antibiotic release contributed to the significant reduction of inherent cytotoxicity of VAN/CDL to cultured fibroblasts. Indeed, all tested concentrations of antibiotics in the free form solution were cytotoxic, in contrast to loaded GG‐based spongy‐like hydrogels. This finding is of great significance, indicating that the proposed DDS can minimize antibiotic induced toxicity. And, considering the intended local application, toxicity of antibiotics can be circumvented, not only systemically but also at the site of infection.^63^

After diffusion from loaded GG‐based spongy‐like hydrogels, antibiotics remained active against MRSA, as suggested by the similar in vitro antibacterial effect as free VAN/CLD solutions, and further corroborated through the in vivo mouse model of diabetic infected wounds. In fact, treating MRSA‐infected wounds with loaded GG‐based spongy‐like hydrogel caused a significant reduction of wound bacterial burden, even though insufficient to eradicate infection, once bacterial load was still superior to Log_10_ CFU >5 (clinical threshold for infection).^63^ In addition, a continuous infiltration of inflammatory cells, mostly neutrophils, was observed. And, although this inflammatory infiltrate was apparently less exacerbated in treatment group than in controls, no signs of proliferation or wound closure were detected throughout the experimental period, which may suggest that wound healing progression could have been compromised by the infection and inflammation state.^63^, ^64^ Nevertheless, histological analysis at later timepoints would be necessary to clarify this hypothesis and to better address the impact of proposed DDS on the progression of wound healing.

Immunological analysis confirmed that inflammation was local, rather than systemic, and persisted from day 3 to day 7 post‐treatment. These data also suggested that treatment did not trigger an exacerbation of the host immune response, corroborating the reported biocompatibility of GG reported in our in vitro studies and by others^65^, ^66^ and indicating that the proposed DDS is safe for local application.

In light of our in vivo findings, it is relevant to consider two experimental factors that could improve the therapeutic effect of the tested DDS, namely: (i) the regimen of antibiotic administration, that in our study comprised a single administration, at a lower dose (approximately, 0.04 mg of VAN and 0.05 mg of CLD, equivalent to 1.5 and 2 mg/kg, respectively) than systemic doses of VAN and CLD currently used in the clinic; and (ii) the absence of wound debridement, that is a key clinical procedure in the treatment of DFI. Wound debridement is crucial to disrupt and remove mature bacterial biofilms, leaving wounds with bacterial forms more susceptible to the action of antibiotics and the immune system, such as planktonic bacteria or even newly formed biofilms.^67^, ^68^, ^69^, ^70^ In agreement, a recent retrospective cohort study reported that combining conservative surgical debridement with the local implantation of an antibiotic‐loaded delivery system promoted an effective and safe resolution of complex foot infections.^71^ Therefore, from the obtained data we can speculate that by increasing the administration dose and frequency of proposed DDS, ideally combined with wound debridement, a better infection control could be achieved.

## 4 MATERIALS AND METHODS

### 4.1 Materials

Merck KGaA (Germany) provided gellan gum (Gelzan™ CM), vancomycin hydrochloride from *Streptomyces orientalis* (CAS number 1404‐93‐9), clindamycin hydrochloride (CAS number 21462‐39‐5), calcium chloride, formic acid, acetonitrile (LC–MS grade), methanol (LC–MS grade), resazurin sodium salt, streptozotocin (STZ), Mueller‐Hinton (MH) broth and MH agar. Mannitol salt agar (MSA) was purchased from Biolife Italiana S.r.l. (Italy). Dulbecco's Modified Eagle Medium (DMEM), fetal bovine serum (FBS), trypsin–EDTA buffer, L‐glutamine and HEPES buffer were purchased from Gibco™, ThermoFisher Scientific (USA). Other reagents, unless otherwise noted, were of analytical grade. LEGENDplex™ multi‐analyte flow assay kit (Cat. No. 740846) was provided by Biolegend® (USA). *S. aureus* Rosenbach BAA 2313™ was provided by American Type Culture Collection.

### 4.2 Checkerboard assay

Antimicrobial interaction between VAN and CLD was assessed in vitro through the checkerboard assay, in a 96‐well plate.^44^ First, two‐fold concentration gradient of antibiotics was obtained in a two‐dimensional manner, starting from a working solution at 8 μg/mL for VAN and 2 μg/mL for CLD. Then, an inoculum of MRSA was prepared by emulsifying overnight colonies from MH agar in saline solution, to a turbidity equivalent to a 0.5 McFarland standard (approximately 10^8^ CFU/mL). This suspension was diluted to 10^6^ CFU/mL in MH broth and plated 100 μL/well. Final antibiotic concentrations ranged 0.031–2 μg/mL for VAN and 0.008–0.5 μg/mL for CLD. Negative (medium only) and positive control (bacteria only) wells were also included. Plate was incubated at 37°C/24 h and afterwards, 10% resazurin was added to each well to facilitate visual reading and left for 3 h at 37°C. Minimal inhibitory concentration (MIC) was determined as the lowest antibiotic concentration resulting in no visible growth. Antimicrobial interaction was calculated as FICI, as following Equation (1):

(1) $$\text{FICI}=\left({\mathrm{MIC}}_{\mathrm{VAN}}\text{in}\;\text{combination}/{\mathrm{MIC}}_{\mathrm{VAN}}\;\text{alone}\right)+\left({\mathrm{MIC}}_{\mathrm{CLD}}\text{in}\;\text{combination}/{\mathrm{MIC}}_{\mathrm{CLD}}\;\text{alone}\right)$$

Antimicrobial interactions were interpreted as synergistic (FICI ≤ 0.5), additive (0.5 < FICI ≤ 1), indifferent (1 < FICI < 4), or antagonistic (FICI ≥ 4).

### 4.3 Preparation of GG‐based spongy‐like hydrogels

GG‐based spongy‐like hydrogels were obtained as previously described.^38^ Briefly, Gelzan™ CM powder was dissolved at 0.75% in distilled water at 90°C under stirring. Afterwards, temperature was decreased to 65°C and aqueous solutions of antibiotics were added to the GG solution, according to Table 1. Subsequently, calcium chloride solution was added (0.03% final concentration) and the hydrogel was rapidly transferred to a petri dish for gelation and ionic crosslinking. After 1 h of stabilization at room temperature, hydrogel discs of 1‐mm thickness and 5‐mm diameter were cut using a punch biopsy, frozen at −80°C for 24 h and freeze‐dried for 3 days (LyoAlfa 10/15, Telstar®, Spain). Dried GG‐based structures incorporating VAN and CLD, referred as loaded GG‐based structures, were stored at 4°C until further usage. Unloaded GG‐based structures were also prepared and used as controls. Spongy‐like hydrogels were attained by hydration of dried structures.

### 4.4 Morphological analysis

Morphology of loaded and unloaded GG‐based structures was analyzed using a high‐resolution field emission scanning electron microscope (SEM, AURIGA Compact, Zeiss, Germany) equipped with energy dispersive electron X‐ray (EDX) spectroscopy (Bruker QUANTAX ESPIRIT 2.0 EDS system, X‐flash detector, Germany). Previously, samples were placed onto metal holders using carbon double‐sided tape and platinum sputter coated (Leica EM ACE600, Leica Microsystems, Germany).

### 4.5 Water uptake and retention capacity

Dried structures were weighed, immersed in PBS (0.01 M, pH 7.4) for 24 h at a temperature close to the skin (33 ± 1°C). At defined timepoints (0.5, 1.5, 3.5, 20, and 24 h), spongy‐like hydrogels were retrieved, tapped with filter paper to remove excess of PBS, and weighed again to estimate water uptake (%) according to Equation (2):

(2) $$\text{Water uptake}\;\left(\%\right)=\frac{W_{t}-W_{i}}{W_{i}}\times 100$$

where *W*_*t*_ is the weight of the spongy‐like hydrogel at a predetermined time (*t*) and *W*_i_ is the initial weight of dried GG structure.

### 4.6 FTIR‐ATR

Possible antibiotic‐polymer molecular interactions were analyzed by FTIR‐ATR using an IR Prestige‐21 spectrometer (Schimadzu, Japan) with an ATR sampling mode. Transmittance spectra of loaded GG‐based structures and raw materials (VAN, CLD, and GG) were recorded at 40 scans in the spectral range of 4400–700 cm^−1^, with a resolution of 4 cm^−1^.

### 4.7 In vitro antibiotic release

The release of antibiotics from loaded GG‐based spongy‐like hydrogels was assessed in vitro, at 33 ± 1°C and 60 rpm, up to 56 h. Loaded GG‐based structures were immersed in 1.2 mL of PBS (0.01 M, pH 7.4) and, at predetermined timepoints, 0.3 mL samples were retrieved. An equivalent volume of fresh PBS was added at each sampling time. The amount of released antibiotics was quantified by liquid chromatography–mass spectrometry (LC–MS), on an Agilent 1260 Infinity II Prime LC system coupled to an Agilent Ultivo triple quadrupole LC/MS with an electrospray ionization (ESI) source (©Agilent Technologies, Inc.).

Chromatographic separation was performed on an Atlantis® C18 column (2.1 × 50 mm, 3 μm) (Waters, USA), at 35°C by gradient elution using 0.1% formic acid in Milli‐Q water (mobile phase A) and 0.1% formic acid in acetonitrile (mobile phase B). The volume of injection was 5 μL and the flow rate was set to 0.4 mL min^−1^, considering the following gradient program (minute, %B): (0.0, 10), (0.5, 10), (4.0, 90), (5.5, 90), (5.6, 10), (8.0, 10). MS was operated in positive ESI mode. Gas flow was 7 L min^−1^ at 305°C, and the sheath gas flow was 11 L min^−1^ at 250°C, respectively. Nebulizer pressure was 15 psi and capillary voltage was 4000 V. Fragmentor and collision energies were optimized to 32 and 25 V, respectively. Ions detection was performed using dynamic multiple reaction monitoring (MRM) mode, according to Table 2.

**TABLE 2:** LC–MS ion acquisition parameters in dynamic MRM mode for VAN and CLD detection.

	VAN	CLD
Precursor ion (*m/z*)	724.9	425.3
Product ion (*m/z*)	144.0	126.1
Fragmentor (V)	25	32
Collision energy (V)	10	25
Retention time (min)	3.78	5.13
Window retention time (min)	1.1	1.1
Average dwell (ms)	499.5	499.5

MRM data were analyzed using Agilent MassHunter Quantitative Analysis software (version 10.0). Total released mass of VAN and CLD was quantified and used to estimate the percentage of cumulative antibiotic release as a function of time (h).

Release data were analyzed by fitting to mathematical models, including zero‐order release kinetics, first‐order release kinetics, Higuchi model and Korsmeyer‐Peppas model, to estimate the mechanism(s) governing antibiotics release. The adjusted coefficient of determination (*R* ^2^_adjusted_) was used as the indicator of the best fitting model.

### 4.8 Antibacterial activity

Broth microdilution and agar diffusion assays were used to assess the antibacterial activity of loaded GG‐based spongy‐like hydrogels. An inoculum was prepared as described for checkerboard assay.

For broth microdilution, inoculum was first diluted in MH broth to 10^6^ CFU/mL and then transferred to respective wells of a 96‐well plate (100 μL/well). For testing loaded GG‐based spongy‐like hydrogels, MH broth (100 μL) was added to respective wells, followed by loaded GG‐based structures. Free VAN/CLD solutions were also tested by dilution with bacterial suspension. Unloaded GG‐based spongy‐like hydrogels were used as material control. Positive (bacteria only) and negative (medium only) growth controls were also used. Plates were then incubated at 37°C for 24 h. Bacterial metabolic viability was assessed using resazurin assay as described for checkerboard assay and measuring optical density at 575 and 610 nm wavelengths (Infinite® 200 microplate reader, Tecan, Switzerland). In parallel, viable colonies were quantified by performing serial 10‐fold dilutions of test wells and further plating on MH agar. After incubation at 37°C for 24 h, CFU were counted and presented as Log_10_CFU.

Regarding agar diffusion assay, 10^8^ CFU of freshly prepared inoculum were seeded on MH agar. Loaded GG‐based structures‐4 and unloaded GG‐based hydrogels (control) were placed on top and hydrated with PBS (5 μL). After incubation at 37°C for 24 h, the zones of bacterial growth inhibition (mm) were measured using a ruler.

### 4.9 Cytocompatibility

Cytocompatibility of loaded GG‐based spongy‐like hydrogels was compared with free VAN/CLD solutions and analyzed by the metabolic activity of mouse fibroblasts L929 (85011425, European Collection of Authenticated Cell Cultures). Cells were cultured in DMEM supplemented with 10% FBS/1% L‐glutamine, in a humidified atmosphere at 37 ± 1°C and 5% CO_2_.

Briefly, cellular suspensions were prepared at 10^5^ cells/mL, seeded in 48‐well plates (100 μL/well) and incubated for 24 h for adhesion. Afterwards, 0.2 mL of culture medium was added and loaded GG‐based structures were placed onto the wells. For free VAN/CLD wells, 0.2 mL of antibiotics solution was added directly. Unloaded GG‐based spongy‐like hydrogels were included as material control, as wells as cells only (positive control) and culture medium only (negative control). Cells were incubated for 3 days at 37 ± 1°C and 5% CO_2_, and then tested for metabolic viability using the resazurin assay as described for broth microdilution assay. Cell viability reduction higher than 30% was considered for cytotoxicity, according with ISO 10993‐5:2009.^72^

### 4.10 Animals

Animals were handled in accordance with the Directive 2010/63/EU of the European Parliament and of the Council, on the protection of animals used for scientific purposes (transposed to Portuguese law Decreto‐Lei 2013/113, August 7). This study was approved by the Institutional Animal Care and Use Committee of University of Minho (ORBEA EM/ICVS‐I3Bs_001/2020) and performed at Life and Health Sciences Research Institute of University of Minho.

Specific pathogen‐free (SPF) male C57BL/6 mice (*n* = 50) were obtained from Charles River Laboratories (Barcelona, Spain) and allowed to acclimatize for at least 1 week in SPF animal facilities before experimentation. Mice were allowed food and water ad libitum and used at 8‐to‐12 weeks old.

Mice were randomly divided in two endpoints groups (3‐ and 7‐days post‐treatment (dpt)), each one containing three experimental groups: (i) treatment (loaded GG‐based hydrogel‐4), (ii) control of formulation (unloaded GG‐based hydrogel), and (iii) control of infection (without any formulation).

### 4.11 Diabetes induction

Type 1 diabetes mellitus was induced by intraperitoneal injection of STZ (50 mg/kg) for 5 consecutive days, as previously.^73^ During the injection period, mice were given 10% sucrose water to prevent fatal hypoglycemia. Nine days after treatment, mice were fasted for 6 h and a blood sample was collected from the tail vein for glucose measurement (FreeStyle Precision Neo, Abbott Laboratories, USA). Glucose levels higher than 150 mg/dL were considered for hyperglycemia.

### 4.12 MRSA inoculation of polycarbonate membranes

Polycarbonate membranes, 0.2 μm pore size (Merck KGaA, Germany) were used for MRSA growth and further wound infection, as previously.^46^ First, membranes were cut in 5‐mm diameter discs and sterilized on both sides by UV light for 30 min. Membranes were then placed on MSA and inoculated with 10^2^ CFU of MRSA, previously diluted from a 10^8^ bacterial suspension prepared as described for checkerboard assay, and incubated overnight at 37°C.

### 4.13 Mouse model of MRSA‐infected wounds

Mouse model of MRSA‐infected wounds was created following our previously optimized protocol.^74^ On the day before surgery, dorsal hair was removed by using a hair clipper and then applying a depilatory cream (Veet). For surgery, mice were anesthetized (75 mg/kg ketamine and 1 mg/kg medetomidine), placed on their side, and dorsal skin was pulled and punched through the folded skin using a 5‐mm biopsy. A silicone splint ring (15‐mm external diameter and 6‐mm internal diameter) was secured around each wound with cyanoacrylate glue and four interrupted sutures of 5/0 nylon to prevent wound contraction.

Wounds were infected with MRSA‐inoculated polycarbonate membranes (approximately 10^9^ CFU), by placing the biofilm in direct contact with the wound bed. Wounds were covered with Durapore™ self‐adhering bandage (3M, USA) and mice were allowed to fully recover from anesthesia under a warming lamp. From the day of surgery and during the following 2 days, analgesia and vitaminic supplementation was subcutaneously administered for postoperative pain relief and hydration of animals. After 2 days, mice received a lower dose of anesthesia, and polycarbonate membranes were removed before treatment application. Finally, a sterile transparent semi‐occlusive dressing Tegaderm (3M, USA) was applied covering the wounds and splints, followed by an Omnifix elastic bandage (Hartmann).

### 4.14 Immunological assays

Quantification of cytokine and chemokine in serum and in wound tissue was performed using the LEGENDplex™ multi‐analyte flow assay kit, according with manufacturer's instructions.

For serum sampling, whole blood was collected from the orbital sinus, centrifuged (2000 g/10 min) and serum was aliquoted and stored at −80°C until usage.

For wound tissue collection, the right wound of each mouse was cut across the midline and one of the half‐portions was immediately frozen in liquid nitrogen and stored at −80°C until further processing for protein extraction. For this purpose, tissue samples were minced by manual grinding in ice‐cold PBS containing 1% EDTA and protease and phosphatase inhibitors. Homogenates were vortexed and centrifuged (13,000 *g*/15 min/4°C). The supernatant containing soluble protein was collected, aliquoted and stored at −80°C until analysis.

### 4.15 Wound bacterial burden quantification

Left wound was harvested, minced in a petri dish, resuspended in PBS and vortexed with 2‐mm glass beads. Ten‐fold serial dilutions of wound homogenates were plated onto MSA and incubated at 37°C for 24 h before CFU quantification. Bacterial burden was expressed as Log_10_CFU/wound.

### 4.16 Histological analysis

Half‐portion of right wound tissue was fixed in 10% neutral buffered formalin (at room temperature and mild stirring, 24 h) and further embedded in paraffin, sectioned (4‐μm thickness) and stained with Hematoxylin and Eosin (H&E) using standard protocols.

### 4.17 Statistical analysis

Data was expressed as mean ± standard deviation (SD) of at least three independent experiments. Normality was tested using Shapiro‐Walk test and, if observed normal distribution and variance homogeneity, One‐way analysis of variance (ANOVA) with Tukey's multiple comparisons test was used. Otherwise, data was analyzed using Kruskal‐Wallis test with Dunn's multiple comparisons test. In vivo data was analyzed using two‐way ANOVA with Sidak's multiple comparison test. Significance levels were set as *p < 0.05, **p < 0.01, ***p < 0.001 and ****p < 0.0001. Analyses were performed using GraphPad Prism 7.0a (GraphPad Software Inc., USA).

## 5 CONCLUSION

We proposed an antibiotic topical delivery system using a GG‐based spongy‐like hydrogel as a therapeutic approach targeting MRSA‐DFI, aiming to maximize therapeutic concentrations at the infection site and to circumvent the current limitations of antibiotic therapy in DFI.

Overall, developed DDS demonstrated attractive features for topical application, particularly the large capacity to absorb biological fluids, while enabling the controlled release of incorporated antibiotics that promoted the reduction of antibiotic‐associated toxicity without impairing their antibacterial activity. Further adjustments of antibiotic dosing regimen, through the increment of VAN/CLD incorporated in the GG‐based spongy‐like hydrogel and the frequency of administration, could contribute to optimize the in vivo efficacy of this DDS.

## AUTHOR CONTRIBUTIONS

**Ana Isabel Mendes:** Conceptualization (equal); formal analysis (equal); investigation (equal); methodology (equal); writing – original draft (equal). **Alexandra Gabriel Fraga:** Conceptualization (equal); investigation (supporting); methodology (supporting); writing – review and editing (equal). **Maria João Peixoto:** Investigation (supporting); writing – review and editing (equal). **Ivo Aroso:** Investigation (supporting); methodology (supporting); writing – review and editing (equal). **Adhemar Longatto‐Filho:** Investigation (supporting); writing – review and editing (equal). **Alexandra Pinto Marques:** Conceptualization (equal); resources (equal); supervision (equal); writing – review and editing (equal). **Jorge Pedrosa:** Conceptualization (equal); resources (equal); supervision (equal); writing – review and editing (equal).

## CONFLICT OF INTEREST STATEMENT

The authors have no conflicts of interest to declare.

### PEER REVIEW

The peer review history for this article is available at https://publons.com/publon/10.1002/btm2.10504.

## Supporting information


**Figure S1.** Serum levels of pro‐inflammatory (KC, IL‐1β, TNF‐α, IL‐23, IL‐18, IL‐6) and anti‐inflammatory (G‐CSF, TARC) mediators from diabetic mouse model of MRSA‐infected wounds after 3 and 7 days of treatment. *p < 0.05; **p < 0.01.

## Data Availability

The data that support the findings of this study are available from the corresponding author upon reasonable request.

## References

[1] Sun H , Saeedi P , Karuranga S , et al. IDF diabetes atlas: global, regional and country‐level diabetes prevalence estimates for 2021 and projections for 2045. Diabetes Res Clin Pract. 2022;183:109119. 34879977 10.1016/j.diabres.2021.109119 PMC11057359

[2] Armstrong DG , Boulton AJM , Bus SA . Diabetic foot ulcers and their recurrence. N Engl J Med. 2017;376(24):2367‐2375. 28614678 10.1056/NEJMra1615439

[3] Cavanagh PR , Lipsky BA , Bradbury AW , Botek G . Treatment for diabetic foot ulcers. Lancet. 2005;366(9498):1725‐1735. 16291067 10.1016/S0140-6736(05)67699-4

[4] Patel S , Srivastava S , Singh MR , Singh D . Mechanistic insight into diabetic wounds: pathogenesis, molecular targets and treatment strategies to pace wound healing. Biomed Pharmacother. 2019;112:108615. 30784919 10.1016/j.biopha.2019.108615

[5] Malone M , Gosbell IB , Dickson HG , Vickery K , Espedido BA , Jensen SO . Can molecular DNA‐based techniques unravel the truth about diabetic foot infections? Diabetes Metab Res Rev. 2017;33(1):7. 10.1002/dmrr.2834 27291330

[6] World Health Organization . Global Report in Diabetes. WHO; 2016.

[7] Lipsky BA , Aragón‐Sánchez J , Diggle M , et al. IWGDF guidance on the diagnosis and management of foot infections in persons with diabetes. Diabetes‐Metabolism Res Rev. 2016;32:45‐74. 10.1002/dmrr.2699 26386266

[8] Olid AS , Solà I , Barajas‐Nava LA , et al. Systemic antibiotics for treating diabetic foot infections. Cochrane Database Syst Rev. 2015;9:113. 10.1002/14651858.CD009061.pub2 PMC8504988 26337865

[9] Citron DM , Goldstein EJC , Merriam CV , Lipsky BA , Abramson MA . Bacteriology of moderate‐to‐severe diabetic foot infections and in vitro activity of antimicrobial agents. J Clin Microbiol. 2007;45(9):2819‐2828. 17609322 10.1128/JCM.00551-07 PMC2045270

[10] Crouzet J , Lavigne JP , Richard JL , Sotto A . Diabetic foot infection: a critical review of recent randomized clinical trials on antibiotic therapy. Int J Infect Dis. 2011;15(9):E601‐E610. 21737333 10.1016/j.ijid.2011.05.003

[11] Silva V , Almeida F , Carvalho JA , et al. Emergence of community‐acquired methicillin‐resistant Staphylococcus aureus EMRSA‐15 clone as the predominant cause of diabetic foot ulcer infections in Portugal. Eur J Clin Microbiol Infect Dis. 2020;39(1):179‐186. 31599357 10.1007/s10096-019-03709-6

[12] Eleftheriadou I , Tentolouris N , Argiana V , Jude E , Boulton AJ . Methicillin‐resistant Staphylococcus aureus in diabetic foot infections. Drugs. 2010;70(14):1785‐1797. 20836573 10.2165/11538070-000000000-00000

[13] Barwell ND , Devers MC , Kennon B , et al. Diabetic foot infection: antibiotic therapy and good practice recommendations. Int J Clin Pract. 2017;71(10):10. 10.1111/ijcp.13006 28892282

[14] Lipsky BA , Berendt AR , Cornia PB , et al. 2012 Infectious Diseases Society of America clinical practice guideline for the diagnosis and treatment of diabetic foot infections. Clin Infect Dis. 2012;54(12):E132‐U232. 22619242 10.1093/cid/cis346

[15] Hasan N , Cao J , Lee J , et al. Bacteria‐targeted clindamycin loaded polymeric nanoparticles: effect of surface charge on nanoparticle adhesion to MRSA, antibacterial activity, and wound healing. Pharmaceutics. 2019;11(5):236. 31096709 10.3390/pharmaceutics11050236 PMC6571677

[16] Cong YG , Yang SJ , Rao XC . Vancomycin resistant *Staphylococcus aureus* infections: a review of case updating and clinical features. J Adv Res. 2020;21:169‐176. 32071785 10.1016/j.jare.2019.10.005 PMC7015472

[17] Smilack JD , Wilson WR , Cockerill FR . Tetracyclines, chloramphenicol, erythromycin, clindamycin, and metronidazole. Mayo Clin Proc. 1991;66(12):1270‐1280. 1749296 10.1016/s0025-6196(12)62479-3

[18] Murphy PB , Bistas KG , Le JK . Clindamycin. 2022 Acessed July 2022. https://www.ncbi.nlm.nih.gov/books/NBK519574/

[19] Gustafson CT , Boakye‐Agyeman F , Brinkman CL . Controlled delivery of vancomycin via charged hydrogels. PLoS One. 2016;11(1):e0146401. 26760034 10.1371/journal.pone.0146401 PMC4711919

[20] Patel S , Preuss C , Bernice F . Vancomycin. 2022 Acessed July 2022. https://www.ncbi.nlm.nih.gov/books/NBK459263/.

[21] Kalita S , Devi B , Kandimalla R , et al. Chloramphenicol encapsulated in poly‐ε‐caprolactone–pluronic composite: nanoparticles for treatment of MRSA‐infected burn wounds. Int J Nanomed. 2015;10(1):2971‐2984. 10.2147/IJN.S75023 PMC4404939 25931822

[22] Dorati R , DeTrizio A , Genta I , et al. An experimental design approach to the preparation of pegylated polylactide‐co‐glicolide gentamicin loaded microparticles for local antibiotic delivery. Mater Sci Eng C. 2016;58:909‐917. 10.1016/j.msec.2015.09.053 26478386

[23] Dzikowski M , Castanié N , Guedon A , Verrier B , Primard C , Sohier J . Antibiotic incorporation in jet‐sprayed nanofibrillar biodegradable scaffolds for wound healing. Int J Pharm. 2017;532(2):802‐812. 28864389 10.1016/j.ijpharm.2017.08.117

[24] Kaur P , Gondil VS , Chhibber S . A novel wound dressing consisting of PVA‐SA hybrid hydrogel membrane for topical delivery of bacteriophages and antibiotics. Int J Pharm. 2019;572:118779. 31740093 10.1016/j.ijpharm.2019.118779

[25] Anjum A , Sim CH , Ng SF . Hydrogels containing antibiofilm and antimicrobial agents beneficial for biofilm‐associated wound infection: formulation characterizations and in vitro study. AAPS PharmSciTech. 2018;19(3):1219‐1230. 29280044 10.1208/s12249-017-0937-4

[26] Roy DC , Tomblyn S , Burmeister DM , et al. Ciprofloxacin‐loaded keratin hydrogels prevent Pseudomonas aeruginosa infection and support healing in a porcine full‐thickness excisional wound. Adv Wound Care. 2015;4(8):457‐468. 10.1089/wound.2014.0576 PMC4505758 26244102

[27] Kumar S , Pal S , Thakur J , et al. Nonimmunogenic hydrogel‐mediated delivery of antibiotics outperforms clinically used formulations in mitigating wound infections. ACS Appl Mater Interfaces. 2021;13:44041‐44053. 34491724 10.1021/acsami.1c12265

[28] Kalita H , Hazarika A , Kalita S , Kandimalla R , Devi R . Antimicrobials tethering on suture surface through a hydrogel: a novel strategy to combat postoperative wound infection. RSC Adv. 2017;7:32637‐32646.

[29] Liang Y , Li M , Yang Y , Qiao L , Xu H , Guo B . pH/glucose dual responsive metformin release hydrogel dressings with adhesion and self‐healing via dual‐dynamic bonding for athletic diabetic foot wound healing. ACS Nano. 2022;16:3194‐3207. 35099927 10.1021/acsnano.1c11040

[30] Hu S , Cai X , Qu X , et al. Preparation of biocompatible wound dressings with long‐term antimicrobial activity through covalent bonding of antibiotic agents to natural polymers. Int J Biol Macromol. 2019;123:1320‐1330. 30248428 10.1016/j.ijbiomac.2018.09.122

[31] Bera H , Mothe S , Maiti S , Vanga S . Carboxymethyl fenugreek galactomannan‐gellan gum‐calcium silicate composite beads for glimepiride delivery. Int J Biol Macromol. 2018;107:604‐614. 28916379 10.1016/j.ijbiomac.2017.09.027

[32] Mahdi MH , Conway BR , Mills T , Smith AM . Gellan gum fluid gels for topical administration of diclofenac. Int J Pharm. 2016;515(1–2):535‐542. 27789369 10.1016/j.ijpharm.2016.10.048

[33] Nair AB , Shah J , Aljaeid BM , Al‐Dhubiab BE , Jacob S . Gellan gum‐based hydrogel for the transdermal delivery of nebivolol: optimization and evaluation. Polymers. 2019;11(10):1699. 31623262 10.3390/polym11101699 PMC6836162

[34] Salunke SR , Patil SB . Ion activated in situ gel of gellan gum containing salbutamol sulphate for nasal administration. Int J Biol Macromol. 2016;87:41‐47. 26899173 10.1016/j.ijbiomac.2016.02.044

[35] Dhanka M , Shetty C , Srivastava R . Methotrexate loaded gellan gum microparticles for drug delivery. Int J Biol Macromol. 2018;110:346‐356. 29223759 10.1016/j.ijbiomac.2017.12.026

[36] Prezotti FG , BSF C , Evangelista RC . Mucoadhesive beads of gellan gum/pectin intended to controlled delivery of drugs. Carbohydr Polym. 2014;113:286‐295. 25256487 10.1016/j.carbpol.2014.07.021

[37] Carmona‐Moran CA , Zavgorodnya O , Penman AD , et al. Development of gellan gum containing formulations for transdermal drug delivery: component evaluation and controlled drug release using temperature responsive nanogels. Int J Pharm. 2016;509(1–2):465‐476. 27260133 10.1016/j.ijpharm.2016.05.062

[38] da Silva LP , Cerqueira MT , Sousa RA , Reis RL , Correlo VM , Marques AP . Engineering cell‐adhesive gellan gum spongy‐like hydrogels for regenerative medicine purposes. Acta Biomater. 2014;10(11):4787‐4797. 25048775 10.1016/j.actbio.2014.07.009

[39] Ferris CJ , Gilmore KJ , Wallace GG , Panhuis M . Modified gellan gum hydrogels for tissue engineering applications. Soft Matter. 2013;9(14):3705‐3711.

[40] da Silva LP , Santos TC , Rodrigues DB , et al. Stem cell‐containing hyaluronic acid‐based spongy hydrogels for integrated diabetic wound healing. J Invest Dermatol. 2017;137(7):1541‐1551. 28259681 10.1016/j.jid.2017.02.976

[41] Muthukumar T , Song JE , Khang G . Biological role of gellan gum in improving scaffold drug delivery, cell adhesion properties for tissue engineering applications. Molecules. 2019;24(24):4514. 31835526 10.3390/molecules24244514 PMC6943741

[42] Cerqueira MT , da Silva LP , Santos TC , et al. Gellan gum‐hyaluronic acid spongy‐like hydrogels and cells from adipose tissue synergize promoting neoskin vascularization. ACS Appl Mater Interfaces. 2014;6(22):19668‐19679. 25361388 10.1021/am504520j

[43] Berti FV , Srisuk P , da Silva LP , Marques AP , Reis RL , Correlo VM . Synthesis and characterization of electroactive gellan gum spongy‐like hydrogels for skeletal muscle tissue engineering applications. Tissue Eng Part A. 2017;23(17–18):968‐979. 28152667 10.1089/ten.TEA.2016.0430

[44] CLSI , ed. Performance Standards for Antimicrobial Susceptibility Testing. 31st ed. . CLSI supplement M100 Clinical and Laboratory Standards Institute; 2021:352.

[45] Zakeri‐Milani P , Loveymi BD , Jelvehgari M , Valizadeh H . The characteristics and improved intestinal permeability of vancomycin PLGA‐nanoparticles as colloidal drug delivery system. Colloids Surf B Biointerfaces. 2013;103:174‐181. 23201735 10.1016/j.colsurfb.2012.10.021

[46] Dimitrovska I , Olumceva T , Markova E , et al. Topical gel with ethyl cellulose based microsponges loaded with clindamycin hydrochloride for acne treatment. Cellulose. 2020;27(12):7109‐7126.

[47] Maver T , Mastnak T , Mihelič M , Maver U , Finšgar M . Clindamycin‐based 3D‐printed and electrospun coatings for treatment of implant‐related infections. Materials. 2021;14(6):1464. 33802712 10.3390/ma14061464 PMC8002500

[48] Mohamed AI , Abd‐Motagaly AME , Ahmed OAA , Amin S , Mohamed Ali A . Investigation of drug‐polymer compatibility using chemometric‐assisted UV‐spectrophotometry. Pharmaceutics. 2017;9(1):7. 28275214 10.3390/pharmaceutics9010007 PMC5374373

[49] Wang B , Adhikari B , Barrow CJ . Highly stable spray dried tuna oil powders encapsulated in double shells of whey protein isolate‐agar gum and gellan gum complex coacervates. Powder Technol. 2019;358:79‐86.

[50] Rufato KB , Souza PR , Oliveira AC , et al. Antimicrobial and cytocompatible chitosan, N,N,N‐trimethyl chitosan, and tanfloc‐based polyelectrolyte multilayers on gellan gum films. Int J Biol Macromol. 2021;183:727‐742. 33915214 10.1016/j.ijbiomac.2021.04.138

[51] Mottola C , Matias CS , Mendes JJ , et al. Susceptibility patterns of Staphylococcus aureus biofilms in diabetic foot infections. BMC Microbiol. 2016;16(1):119. 27339028 10.1186/s12866-016-0737-0 PMC4918071

[52] Dutescu IA , Hillier SA . Encouraging the development of new antibiotics: are financial incentives the right way forward? A systematic review and case study. Infect Drug Resist. 2021;14:415‐434. 33574682 10.2147/IDR.S287792 PMC7872909

[53] Friedman ND , Temkin E , Carmeli Y . The negative impact of antibiotic resistance. Clin Microbiol Infect. 2016;22(5):416‐422. 26706614 10.1016/j.cmi.2015.12.002

[54] Pletz MW , Hagel S , Forstner C . Who benefits from antimicrobial combination therapy? Lancet Infect Dis. 2017;17(7):677‐678. 28442294 10.1016/S1473-3099(17)30233-5

[55] Watanakunakorn C . Mode of action and in vitro activity of vancomycin. J Antimicrob Chemother. 1984;14:7‐18. 10.1093/jac/14.suppl_d.7 6440886

[56] Maddison JE , Watson ADJ , Elliott J . Chapter 8 – antibacterial drugs. In: Maddison JE , Page SW , Church DB , eds. Small Animal Clinical Pharmacology. 2nd ed. W.B. Saunders; 2008:148‐185.

[57] Kim MH . Nanoparticle‐based therapies for wound biofilm infection: opportunities and challenges. IEEE Trans Nanobiosci. 2016;15(3):294‐304. 10.1109/TNB.2016.2527600 PMC4939068 26955044

[58] Xiong MH , Bao Y , Yang XZ , Zhu YH , Wang J . Delivery of antibiotics with polymeric particles. Adv Drug Deliv Rev. 2014;78:63‐76. 24548540 10.1016/j.addr.2014.02.002

[59] Posadowska U , Brzychczy‐Wloch M , Pamula E . Injectable gellan gum‐based nanoparticles‐loaded system for the local delivery of vancomycin in osteomyelitis treatment. J Mater Sci Mater Med. 2016;27(9). 10.1007/s10856-015-5604-2 PMC4666281 26621310

[60] Li A , Khan IN , Khan IU , Yousaf AM , Shahzad Y . Gellan gum‐based bilayer mucoadhesive films loaded with moxifloxacin hydrochloride and clove oil for possible treatment of periodontitis. Drug Des Devel Ther. 2021;15:3937‐3952. 10.2147/DDDT.S328722 PMC8453438 34556975

[61] da Silva LP , Pirraco RP , Santos TC , et al. Neovascularization induced by the hyaluronic acid‐based spongy‐like hydrogels degradation products. ACS Appl Mater Interfaces. 2016;8(49):33464‐33474. 27960396 10.1021/acsami.6b11684

[62] Srisuk P , Berti FV , da Silva LP , Marques AP , Reis RL , Correlo VM . Electroactive gellan gum/polyaniline spongy‐like hydrogels. ACS Biomater Sci Eng. 2018;4(5):1779‐1787. 33445334 10.1021/acsbiomaterials.7b00917

[63] Versey Z , Nizer WSC , Russel E , et al. Biofilm‐innate immune interface: contribution to chronic wound formation. Front Immunol. 2021;12:648554. 33897696 10.3389/fimmu.2021.648554 PMC8062706

[64] Leaper D , Assadian O , Edmiston CE . Approach to chronic wound infections. Br J Dermatol. 2015;173(2):351‐358. 25772951 10.1111/bjd.13677

[65] Ahmad S , Ahmad M , Manzoor K , Purwar R , Ikram S . A review on latest innovations in natural gums based hydrogels: preparations and applications. Int J Biol Macromol. 2019;136:870‐890. 31226381 10.1016/j.ijbiomac.2019.06.113

[66] Oliveira JT , Martins L , Picciochi R , et al. Gellan gum: a new biomaterial for cartilage tissue engineering applications. J Biomed Mater Res A. 2010;93A(3):852‐863. 10.1002/jbm.a.32574 19658177

[67] Attinger C , Wolcott R . Clinically addressing biofilm in chronic wounds. Adv Wound Care. 2012;1(3):127‐132. 10.1089/wound.2011.0333 PMC3839004 24527292

[68] Schultz G , Bjarnsholt T , James GA , et al. Consensus guidelines for the identification and treatment of biofilms in chronic nonhealing wounds. Wound Repair Regen. 2017;25(5):744‐757. 28960634 10.1111/wrr.12590

[69] Atkin L , Bućko Z , Montero EC , et al. Implementing TIMERS: the race against hard‐to‐heal wounds. J Wound Care. 2019;28(3):S5‐S50. 10.12968/jowc.2019.28.Sup3a.S1 30835604

[70] Falcone M , Angelis BD , Pea F , et al. Challenges in the management of chronic wound infections. J Global Antimicrob. 2021;26:140‐147. 10.1016/j.jgar.2021.05.010 34144200

[71] Morley R , Rothwell M , Stephenson J , McIlvenny L , Webb F , Barber A . Complex foot infection treated with surgical debridement and antibiotic loaded calcium sulfate—a retrospective cohort study of 137 cases. J Foot Ankle Surg. 2022;61(2):239‐247. 34364760 10.1053/j.jfas.2021.07.014

[72] ISO 10993‐5:2009 . Biological Evaluation of Medical Devices — Part 5: Tests for In Vitro Cytotoxicity. Internation Organization for Standardization; 2009.

[73] Furman BL . Streptozotocin‐induced diabetic models in mice and rats. Curr Protocol Pharmacol. 2015;70:5.47.1‐5.47.20. 10.1002/0471141755.ph0547s70 26331889

[74] Mendes AI , Peixoto MJ , Marques AP , et al. An optimized mouse model of *Staphylococcus aureus* infected diabetic ulcers. BMC Res Notes. 2022;15(1):293. 36071445 10.1186/s13104-022-06170-5 PMC9450231

