# Supplementary material for: Gellan gum spongy‐like hydrogel‐based dual antibiotic therapy for infected diabetic wounds
Source: Bioeng Transl Med. 2023 Mar 21;8(3):e10504. doi: 10.1002/btm2.10504 (PMC10189450; doi:10.1002/btm2.10504)
Supplement: Supplementary file 1 — Figure S1. Serum levels of pro‐inflammatory (KC, IL‐1β, TNF‐α, IL‐23, IL‐18, IL‐6) and anti‐inflammatory (G‐CSF, TARC) mediators from diabetic mouse model of MRSA‐infected wounds after 3 and 7 days of treatment. *p < 0.05; **p < 0.01. [file BTM2-8-e10504-s001.docx]

Supporting Information

# Gellan gum spongy-like hydrogel-based dual antibiotic therapy for infected diabetic wounds

Ana Isabel Mendes, Alexandra Gabriel Fraga, Maria João Peixoto, Ivo Aroso, Adhemar Longatto-Filho, Alexandra Pinto Marques, Jorge Pedrosa*


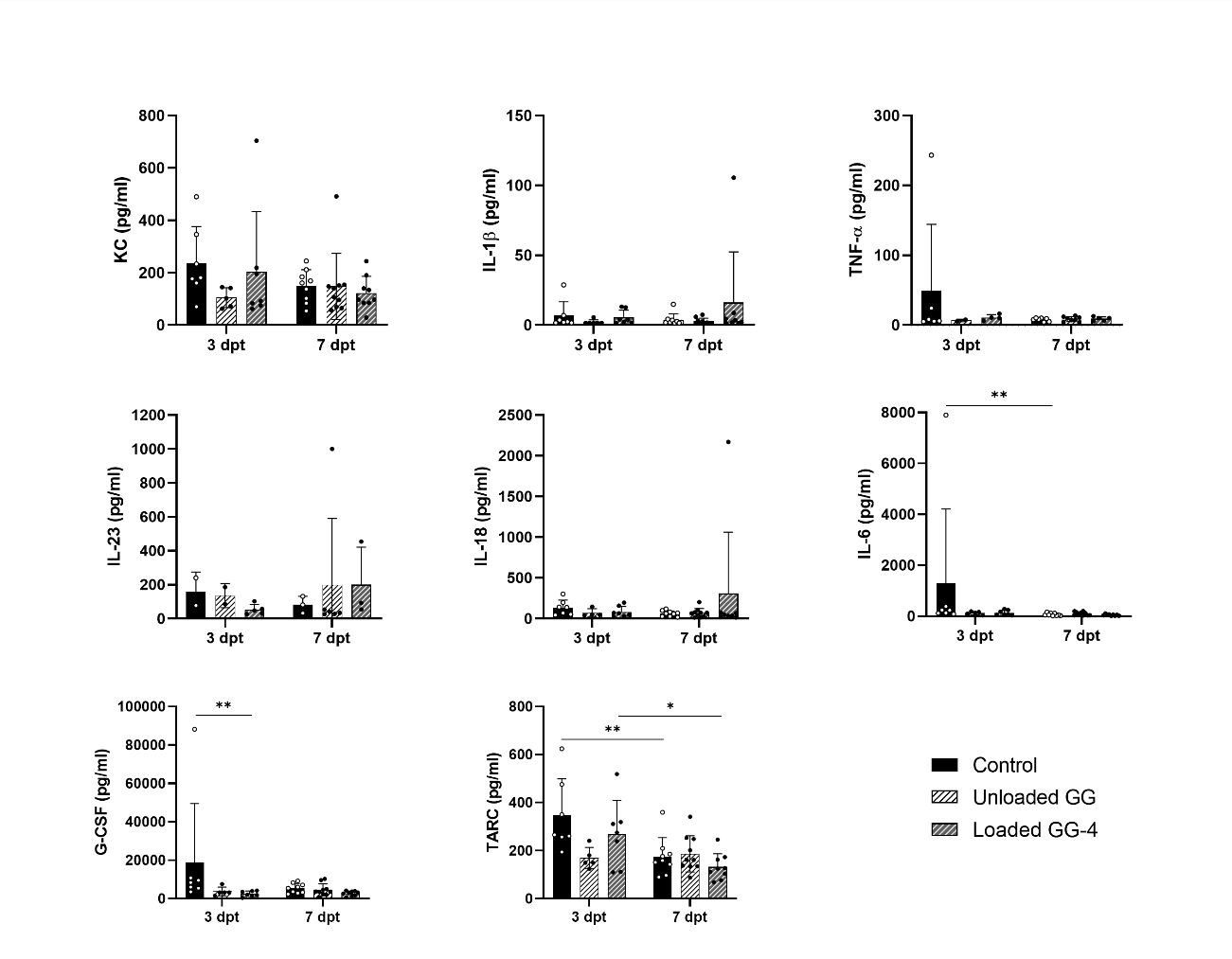


Figure S1. Serum levels of pro-inflammatory (KC, IL-1β, TNF-α, IL-23, IL-18, IL-6) and anti-inflammatory (G-CSF, TARC) mediators from diabetic mouse model of MRSA-infected wounds after 3 and 7 days of treatment. **p*<0.05; ***p*<0.01.
